# Supporting Teams with Designing for Dissemination and Sustainability: the Design, Development, and Usability of a Digital Interactive Platform

**DOI:** 10.21203/rs.3.rs-4276919/v1

**Published:** 2024-05-28

**Authors:** Maura Kepper, Allison L’Hotta, Thembekile Shato, Bethany M. Kwan, Russell E. Glasgow, Douglas Luke, Andrea K. Graham, Ana A. Baumann, Ross C. Brownson, Brad Morse

**Affiliations:** Washington University In St Louis: Washington University in St Louis; University of Colorado School of Medicine: University of Colorado Anschutz Medical Campus School of Medicine; Washington University In St Louis: Washington University in St Louis; University of Colorado School of Medicine: University of Colorado Anschutz Medical Campus School of Medicine; University of Colorado School of Medicine: University of Colorado Anschutz Medical Campus School of Medicine; Washington University In St Louis: Washington University in St Louis; Northwestern University Feinberg School of Medicine; Washington University in St Louis School of Medicine; Washington University In St Louis: Washington University in St Louis; University of Colorado School of Medicine: University of Colorado Anschutz Medical Campus School of Medicine

**Keywords:** Designing for Dissemination, Sustainability, Co-Design, User-Centered Design, Capacity Building, Equity

## Abstract

**Background:**

The use of Designing for Dissemination and Sustainability (D4DS) principles and methods can support the development of research products (interventions, tools, findings) to match well with the needs and context of the intended audience and setting. D4DS principles and methods are not well-known or used during clinical and public health research; research teams would benefit from applying D4DS. This paper presents the development of a new digital platform for teams to learn and apply a D4DS process to their work.

**Methods:**

A user-centered design (UCD) approach engaged users (n=14) and an expert panel (n=6) in an iterative design process from discovery to prototyping and testing. We led five design sessions using Zoom and Figma software over a 5-month period. Users (71% academics; 29% practitioners) participated in at least 2 sessions. Following design sessions, feedback from users were summarized and discussed to generate design decisions. A prototype was then built and heuristically tested with 11 users who were asked to complete multiple tasks within the platform while verbalizing their decision-making using the ‘think aloud’ procedure. The System Usability Scale (SUS) was administered at the end of each testing session. After refinements to the platform were made, usability was reassessed with 7 of 11 same users to examine changes.

**Results:**

The interactive digital platform (the D4DS Planner) has two main components: 1) the Education Hub (e.g., searchable platform with literature, videos, websites) and 2) the Action Planner. The Action Planner includes 7 interactive steps that walk users through a set of activities to generate a downloadable D4DS action plan for their project. Participants reported that the prototype tool was moderately usable (SUS=66) but improved following refinements (SUS=71).

**Conclusions:**

This is a first of its kind tool that supports research teams in learning about and explicitly applying D4DS to their work. The use of this publicly available tool may increase the adoption, impact, and sustainment of a wide range of research products. The use of UCD yielded a tool that is easy to use. The future use and impact of this tool will be evaluated, and the tool will continue to be refined and improved.

## Background

Dissemination and sustainability are two primary pillars of dissemination and implementation (D&I) science. These concepts focus on sharing and maintaining use of effective interventions over time to enhance equitable impact on health at the population level. Active and intentional dissemination efforts help spread interventions to the right people (i.e., the intended audience) in a way that best meets their needs and preferences.(1) The impact of a research initiative is enhanced through active dissemination – spreading the products of research (e.g., interventions) to potential adopters and influencers using planned strategies and appropriate channels. A common goal of researchers is for effective interventions to be used widely (i.e., scaled-up) in routine practice. To maximize impact, researchers also seek to sustain their intervention, defined as the ability to maintain the intervention and its benefits over time.(2–4) While dissemination and sustainment are critical to maximize the impact of research on health and well-being (5), these initiatives can require significant resources and specialized expertise. Funding organizations are beginning to highlight the value of research initiatives focusing on dissemination and sustainability by requiring dissemination plans in grant applications and publishing calls for research focused on sustainability.

Designing for dissemination and sustainability (D4DS) is a new area to D&I science (5–8). D4DS incorporates principles (guiding beliefs based on an approach) from multiple disciplines (e.g., implementation science, communication, business & marketing, systems science, user-centered design). Using transdisciplinary methods (ways of enacting principles from multiple and diverse disciplines) to engage key partners from the start in product development, as well as early and active dissemination and sustainability planning, is not commonly done. According to Kwan et al.,(5) D4DS includes three key components: the research product, dissemination plan to support adoption, and sustainability plan to facilitate use over time. A research ‘product’ is the innovation a research team is trying to disseminate and sustain, which may include an (evidence based) intervention, evidence, treatment, technology, model of care, policy, guideline, or implementation strategy.(5) Three central principles of D4DS, include: 1) beginning with the end in mind when planning research initiatives (i.e., who will adopt, implement, sustain, and potentially benefit from the products of research); 2) ensuring innovation-context fit (i.e., that the products of research can be implemented and equitably effective in real-world settings); and 3) planning for active dissemination and sustainability.(5, 9) When a D4DS approach is not used, a research initiative may suffer from poor innovation-context fit, making it less likely that a research product will be adopted or sustained over time, lessening the impact of the research. A mismatch occurs when a research product – or dissemination and sustainability plans – are not aligned with the priorities, resources, or capacity of the organization, setting, or target audience. (10, 11) The Fit to Context Framework for Dissemination and Sustainability was recently introduced as a process framework to guide research teams through using a D4DS process defined as iteratively using a series of methods to operationalize D4DS principles. (5)

The Fit to Context Framework (F2C) for D4DS aims to support research product adoption, sustainment, and equitable health impacts. The framework includes four phases: conceptualization, design, dissemination, and impact.(5) In the first phase, conceptualization, the objectives are to establish partnerships, identify relevant evidence, and assess the context, in order to establish need and demand for an innovation as well as capacity for change. The second phase, design, includes co-design with users of an innovation (including packaging for use in real-world settings) and dissemination and sustainability plans. In D4DS the responsibility for active dissemination, the third phase, is positioned within the research team and their partners, institutions, and funders. Active dissemination involves deploying the dissemination plan, which specifies the intended audience (who you are trying to reach), the message (what information or product is being disseminated), and the communication channel (how and by whom the message will be shared).(8) The fourth and final phase, impact, examines research product adoption, implementation, sustainment, and broader impact on health and health equity. (5) Health equity is defined as having a fair and just opportunity to be as healthy as possible.(12) D4DS principles could help promote health equity by engaging historically marginalized populations in developing effective interventions and products that meet the needs of these potential users and using strategies that make effective interventions equally accessible in their contexts to generate equal opportunities for health. This may only be realized if the D4DS process is conducted using equity principles (e.g., including the marginalized population, gatekeepers, and diverse groups in developing your product, dissemination, and sustainability plans, using principles of reflexivity and community engagement). We acknowledge that D4DS may not address the scope of obstacles historically marginalized populations face, such as poverty, discrimination, powerlessness, quality education and housing, yet, based on evidence from participatory methods, conducting a D4DS process using equity principles may promote health equity.(13–17) At every stage of the D4DS process there is iterative evaluation and adaptation to ensure “fit to context” – that increases the change that the innovation will be feasible, sustainable, effective, and equitable in real-world settings for diverse populations.

Our purpose was to develop a digital platform, the D4DS Planner, to help clinical and public health researchers and research teams learn about D4DS principles and methods and to operationalize the objectives of the F2C Framework’s conceptualization and design phases. To design the D4DS Planner, we engaged potential users in a participatory design process using user-centered design methodology. User-centered design provides a set of methods for eliciting user perspectives, preferences and ideas to co-design technologies.(18) This methodology is based on the premise that users are significant and useful partners in the knowledge-production and development process.(19, 20) Participatory design has been increasingly used as a method for the design of health technologies to empower users by involving them in the development and to ensure tools are more likely to be engaging and effective.(19, 21, 22) This paper describes the development of the D4DS planner via participatory design methods, its features and functions, the usability testing of the platform, and future directions for the D4DS planner.

## Methods

### Overview and Guiding Design Framework

**Figure 1:**
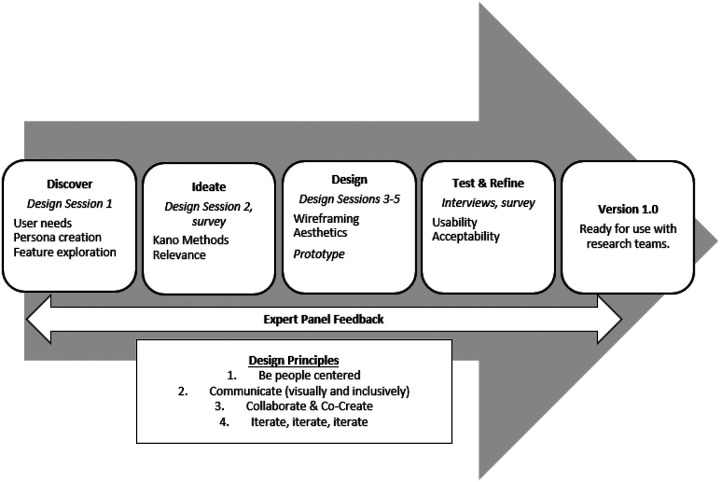
Participatory Design Process for the D4DS Planner

A participatory design approach engaged users and an expert panel in a mixed methods (qualitative and quantitative) iterative user-centered design process starting from discovery to design and testing (Figure 1). We utilized common design principles throughout this process that focused on: 1) being person centered; 2) communicating visually and inclusively; 3) collaborating and co-creating; and 4) iterating.(23, 24) We conducted five 90-minute design sessions conducted over Zoom from September 2022 to February 2023 (Table 1). All design sessions were conducted using Mural(25) or Figma(26), collaborative platforms that allowed users to contribute written artifacts (i.e., responses) during the session. FigJam, a tool in Figma, was used to draw collaboratively during design sessions using an online whiteboard. Following design sessions, qualitative data were summarized and merged with written artifacts (e.g., a sticky note contributed on the mural board) from design sessions or survey results. Weekly team meetings were used to reach consensus on design impacts (i.e., how we would incorporate user feedback into design features of the D4DS planner).

Once a working-version prototype tool was created, usability testing was conducted with a separate group of users who were not familiar with the tool and expert panel members using a combined think-aloud and survey-based approach. The prototype was revised and usability was re-assessed by the same users. Details on the methods for data collection, analysis and results for each design session and usability testing are presented below in the Design Process and Results section. The project was approved as an exempt study by the Washington University in St. Louis Institutional Review Board (#202207165).

### Participants and Recruitment

Users included researchers with experience conducting a health-related project, including academics (e.g., researchers, students, project managers) and clinical and public health practitioners (e.g., clinicians, health department employees). Using purposive and snowball sampling, we recruited 14 users (71% academics and 29% practitioners) to participate as co-designers. Email invitations sent out to users included a brief description of the project goals and D4DS principles. Each co-designer was asked to participate in at least two design sessions. Co-designers reported varying levels of D&I knowledge prior to starting the design sessions, with 21% (n=3) reporting below average, 43% (n=6) reporting average, 36% reported above average knowledge.

The expert panel consisted of academic faculty members (n=6) who are leaders in D&I science, including the developers of the F2C Framework, across three academic institutions. Collectively, the expert panel focuses on public health research in both clinical and community settings and has expertise in the development, implementation, and evaluation of digital health tools. The expert panel members were engaged separately from users in a subset of the design exercises and usability testing.

### Design Process and Results:

#### Design Session 1: Discover

This first design session engaged 6 co-designers to identify the need and demand for key issues this tool should address and explore potential features that will address these needs. We started this design session with a brief overview of D4DS principles and then conducted five design exercises (DE). Co-designers started the session by adding sticky notes to free list: 1) how the tool could benefit and empower users (DE 1.1) and 2) who might benefit from using this tool (DE 1.2). Next, we conducted a persona creation and value proposition generation exercise (DE 1.3). Personas are archetypes of different users who could use the tool and has been used in design to ensure diverse perspectives are accounted for in product design. Therefore, in our sessions, we asked co-designers to consider the perspectives of other potential users (listed in DE 1.2) using a template value proposition stated (i.e., I’m a (user type), who uses the D4DS web tool to (use case) to define (impact)). We then asked co-designer to free list potential features (i.e., ways a user may interact with the content and experience learning in the tool (DE 1.4). Free listing features is a fast way to generate a lot of ideas in a short period of time. (27) Lastly, co-designers placed the listed features into a prioritization bullseye and discussed their ideas by giving 2 minute presentations on their favorite ideas (DE 1.5).

#### Results: Design Session 1

The overall design impact of the first design session was that the tool should increase the transparency of D4DS and accessibility to multiple audiences, and foster collaboration. The tool is intended to help users plan their research and have D4DS as a key principle in their research from the start. To provide education, users suggested the tool contain a repository of key references and resources for individuals engaging in D4DS work. Users felt the tool should provide methods and strategies for engaging community partners in D4DS work. When asked who would benefit from the tool (DE 1.2), there was an emphasis on providing resources not only for people with D&I background, but also the people they partner with, whether that’s a site champion, an individual implementing an intervention, or a software developer designing materials for dissemination. While co-designers felt the primary users were researchers (with various level of D&I knowledge/background), they felt strongly that the tool would facilitate conversation with other partners. Other users may include community members and organizations, practitioners (e.g., clinicians, public health), policy makers, commercial partners, and funders. The main impact of the tool (DE 1.3) was described as: 1) enhancing co-design processes, as well as dissemination and implementation research; 2) increasing understanding of D4DS for multiple audiences; and 3) improving the design, effectiveness and sustainability of research products. The users free-listed (DE 1.4) 35 features that were grouped into 5 categories: grant proposal resources, methods/tools, dissemination resources, educational materials, and general format ideas. The research team combined similar features to generate a final list of 25 features (Table 2), which was used as input for the next design session to prioritize features.

#### Design Session 2: Ideate

The second design session focused on further exploring and prioritizing features. Using information from design session 1, we utilized the Kano Model of Customer Satisfaction exercise for prioritization for the 25 features (Table 2).(28, 29) The Kano Model classifies features as Must-have (i.e., I expect it and would be dissatisfied without it), One-dimensional (i.e., I expect it), Attractive (i.e., I like it), and Indifferent (i.e., I’m neutral). Co-designers and expert panel members completed the Kano as an electronic (REDCap) quantitative survey during or outside the session (DE 2.1).(28) After completing the Kano survey, the 3 zoom participants performed an exercise to free list any other feature ideas and identify their favorite feature from the Kano survey (DE 2.2). Co-designers then grouped features into content areas for learning (DE 2.3) and prioritized the most important content areas to identify which ideas are most important to users (DE 2.4).

##### Results: Design Session 2

A total of 17 participants completed the Kano survey. Table 2 lists the features in order of prioritization according to the Kano survey responses. We identified 1 Must-have feature, 17 Attractive features, and 7 Indifferent features. The Must-have feature was URL links to other content that falls within the discipline/field of D4DS, especially dissemination and sustainability. The number of Attractive features was a promising finding give the many potential opportunities to delight users (a key goal of design), with a relative low risk of dissatisfaction if the feature was not integrated at all. As for the Indifferent features, these features would not yield either satisfaction or dissatisfaction for our users. As a result, the Indifferent features occupied the bottom of our development prioritization, and ultimately were not incorporated into version 1.0 of the web tool.

#### Design Sessions 3–5: Wireframing

The next 3 design sessions focused on iteratively envisioning and creating wireframes of the features that were prioritized in the previous design session. Wireframes are illustrations of a product that are not yet built and typically lack functionality but represent the interface and its intended features and functionalities. The iterative design process allowed users to draw and give feedback on design options (e.g., wireframes) in successive versions. During design session 3, 4 co-designers ideated and drew 1) account creation, 2) a questionnaire that would allow users to set-up a project and guide them on how best to use the tool, 3) a roadmap or visual process that walks the users through the D4DS process, 4) and the landing page. In design session 4, 4 co-designers provided feedback on two versions of the roadmap and sketched a final version of the roadmap. We also discussed which features co-designers wanted to be present on each page that derives from the roadmap and sketched the layout of a single page. In design session 5, co-designers provided comments on a refined version of the single page sketch, sketched the layout of the education hub and spent time discussing the name of the tool and each feature.

#### Results: Design Sessions 3–5

**Figure 2:**
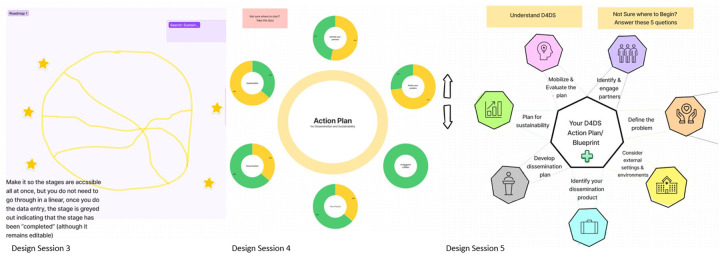
lteration of roadmap wireframe from design sessions 3–5

Co-designers felt that users should have the option to use the tool as a guest or with an account, making it clear to users the benefits of having a login (e.g., saving data, returning later to update work). Discussion identified that the login should be simple but make users feel that the information they add into the tool is secure. A major focus of our prototyping sessions was brainstorming and drawing the feature of a roadmap that would help users walk through a D4DS process. The wireframe of this iterated in each session (Figure 2), with co-designers realizing they did not want this to feel like a linear process. Co-designers were critical in the process of developing easy to understand, action-oriented language to communicate different features of the tool (e.g., D4DS Planner, action planner, action item, cue to equity).

#### Usability Testing: D4DS Planner Prototype

Following all design sessions, our team delivered low-fidelity wireframes and features to a software development company (HICAPPS, https://hicapps.com) that has expertise in developing health-related tools. Our team collaborated with HICAPPS on an iterative build of a prototype of the D4DS Planner to ensure that all feedback from design sessions was incorporated.

In the usability phase, test users were asked to use the D4DS web tool on a laptop computer in a private space in-person or via zoom. The test users were given a general description of the web tool but were not given explicit instructions on how the web tool operated or how it was designed to be used for developing a dissemination and sustainability plan. Test users were asked to carry out two out of a possible three heuristic tasks, including: 1) familiarize yourself with the web tool and figure out the purpose and function of the web tool, 2) create an account, and 3) set up a project to create a D4DS action plan.

To assess the heuristic usability data, an affinity grouping (30, 31) exercise was conducted in Mural, an online collaboration software app, by four team members (MMK, BM, TS, AL) who were integrated into the design and development of the web tool. The research team analyzed internal notes taken during the usability sessions and re-watched recordings of users navigating the tool. Summary phrases were extracted and typed on Mural’s “sticky notes” and mapped together based on similarities in relation to four categories and the collaborator type, e.g., researchers, practitioners, and leadership. The four categories were as follows: (1) What works well in the D4DS web tool? (2) What is not working well, or what are the significant pain points, in the D4DS web tool? (3) What changes do users want to see in the D4DS web tool? (4) Other comments that pertain to the usability of the D4DS web tool. At the end of the usability sessions, test users completed the ten item System Usability Scale (SUS) to quantitatively measure perceived usability of the tool.(32) Usability results were used to refine the prototype tool. Usability was retested on the refined version one of the D4DS planner among the same users who participated in the first round of usability testing. During this round of testing, five questions (reported on a 5-point Likert scale) about the usefulness and appropriateness of the tool were added to the survey.

## Results

### Usability Results

Usability testing on the prototype tool was conducted with a separate group of 11 users (55% academics and 45% clinical and public health practitioners) who were not familiar with the tool using a combined think-aloud and survey-based approach. Five members from the expert panel assembled at the start of the design process also participated in the think-aloud testing only. The affinity grouping synthesis of qualitative usability data is presented in Table 3. Overall, users across the three groups (academics, practitioners, and experts) expressed that the prototype web tool had a professional “look and feel” and contained action-oriented content not often considered when planning for dissemination and sustainability. Users scored the prototype tool a 66 using the SUS, which is slightly below the standard cut-point of 68 to indicate above average usability.(33) While the web tool contained a lot of high-level content, some users felt that there were too many words on the screen. Our main challenge was to provide the user with enough information, while not over-burdening the user who might become reluctant to use the web tool because of its relative complexity. In general, these results were helpful in allowing our team to identify areas of the prototype web tool that needed to be revised to achieve a balance of providing guidance and not overly saturating the interface with directions and jargon. We curated a list of changes that were communicated to the developers of the web tool (**Supplementary Material 2**) and made several wording changes to content to reduce complexity and breadth. Some of the major changes included: 1) the addition of instructional videos and a guided tour that show users the key features of the tool upon first logging in; 2) reduction of text and simplifying language; 3) simplification of the login and project set-up; and 4). Following these updates, usability was reassessed on the first version of the D4DS Planner (presented below) by 7 of the 11 users (57% academics and 43% practitioners) who participated in the first round of usability testing. After refinements, usability scored 71, which was a 5-point increase in the score moving the tool to be perceived as above average for usability. Overall, users felt the tool was useful and appropriate with a mean score of 4.1 (SD 1.1; Table 4).

### The D4DS Planner

**Figure 3:**
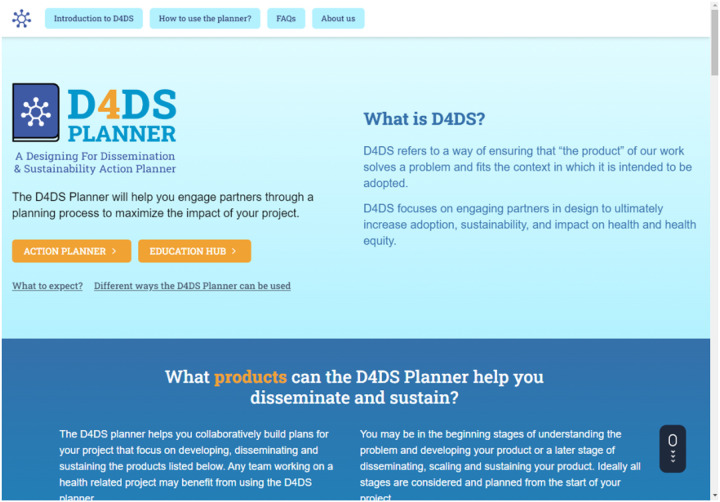
D4DS Planner Home Page

The D4DS Planner is a digital platform designed to help users collaboratively apply D4DS principles and methods to a research project to maximize the potential of their work. The D4DS Planner has two main components: 1) the Education Hub (i.e., a searchable platform with literature, videos, websites, etc.) and 2) the Action Planner that can be accessed from the homepage (Figure 3; d4dsplanner.com).

The Education Hub is available to users without logging in and includes resources (e.g., websites, journal articles, presentations and videos) to educate users on D4DS principles and methods. The Education Hub has search and filter functionality that allow users to find materials related to each D4DS step (called action items) and materials that provide methods, case examples and cues to increase focus on equity. The Action Planner allows users to interact and collaborate with a team in real-time or asynchronously. Users are required to create an account before setting up a project so that all information is saved and can be downloaded as a D4DS Action Plan. The D4DS Action Plan is a detailed document outlining users input from each activity that can be used to support D4DS and application of the F2C Framework. **Supplementary Material 1** provides an example of a D4DS Action plan. It includes information specific to the team’s project that can be used to develop a grant proposal, communicate the value of your work, and justify funding.

**Figure 4:**
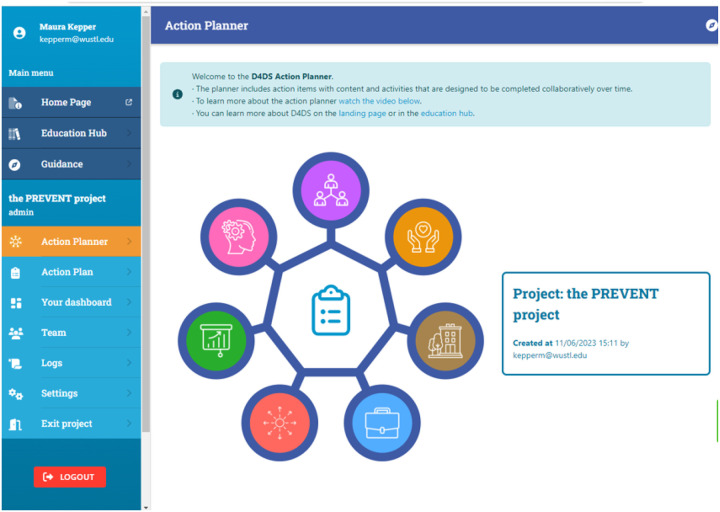
D4DS Action Planner

The Action Planner (Figure 4) is composed of 7 “Action Items” based on the Fit to Context Framework’s conceptualization and design phases (5) that guide users through the D4DS process. Each action item includes an instructional video, interactive activities, educational content, and cues to equity. The cue to equity section prompts users to be intentional about how they are conducting their D4DS activities with the goal of developing equitable products (e.g., those that meet the needs of marginalized populations) that can be equitably accessed by the intended population over time. Examples of cues to equity, include: 1) “Consider who is present and, perhaps most importantly, who is absent in your studies and research team;” 2) “be attentive as to how you ask the questions and attend to issues of power, and of the implicit and explicit assumptions of your questions;” and 3) “think about ways to share information that reflect the values and cultures of your partners.” Each cue is relevant to the activity the team may conduct within the action item and provides further educational materials to help them operationalize the cue.

**Figure 5:**
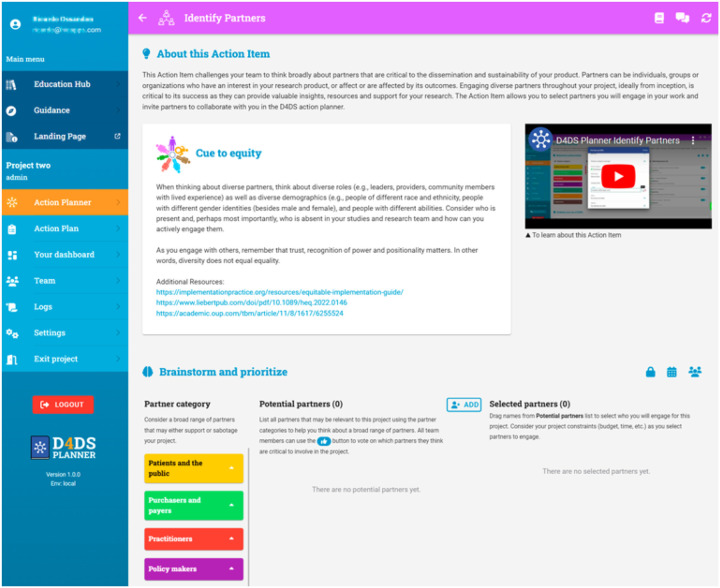
Identify Partners Action Item in the D4DS Planner

Although the tool was built for researchers and research teams, critical to D4DS and strongly valued by co-designers were features that facilitated the engagement of community-based partners, including community members, practitioners, public health organizations, policy makers, etc. The tool invites users to brainstorm and invite relevant partners in the “Identify Partners” Action Item (Figure 5) to log-in and collaborate with them through the process. The tool includes features such as the ability to assign team members specific tasks with a due a date and chat features that foster collaboration. As suggested by co-designers, this process does not need to be completed in it’s entirety or linearly, although some steps do build from others. The tool includes a guidance questionnaire that was co-designed to help users identify which action items are most relevant to their project. The 7 Action Items are:

- **Identify Partners:** This Action Item challenges users to think broadly about partners that are critical to the dissemination and sustainability of their product using the 7 P’s Framework for stakeholder identification in outcomes and effectiveness research.(34) Partners can be individuals, groups or organizations who have an interest in the research product, or affect or are affected by its outcomes. As started above, the Action Item allows you to select partners you will engage in your work and invite partners to collaborate with you in the D4DS action planner.
- **Empathize and Outline the Problem:** Users will engage partners to understand the problem from their perspective and generate a Value Proposition that clearly communicates how their product meets the needs of their target audience. In research, a value proposition can be used to communicate the value of our research to our partners, funding agencies, and the general public.
- **Understand The Context:** This Action Item includes key questions to help users think broadly about characteristics of the people, relationships, product, organization, and environment that may influence the ability to reach the target audience and sustain impact. The goal is to help users consider the multilevel nature of how context can impact how they share, adopt, use and benefit from the product over time.
- **Confirm and Co-design Your Product:**This Action Item allows users to select methods for codesigning their product and packaging it for use in real-world settings. Using co-design methods to engage key partners in the design of a product increases the likelihood that your product will be perceived as feasible, acceptable, useful and equitable.
- **Develop a Dissemination Plan:**This Action Item helps users generate a plan for how to share their product with key audiences, especially those outside of academia. In this action item, the team and their partners will brainstorm all the possible ways they may share about their product, prioritize which methods are the most feasible and will have the most impact, and generate a plan.
- **Plan For Sustainability:** This Action Item helps the team discuss and prioritize what is needed to sustain their product over time and create a practical action plan. The goal is for the team to think early and actively plan for sustainability to enhance the long-term impact of their work.
- **Evaluate Iteratively:** This Action Item helps users evaluate their use of D4DS and develop plans for evaluating dissemination and sustainability. In this Action Item, the tool helps users develop evaluation plans to maximize the intended impact of their dissemination and sustainability plans. Evaluation should be conducted iteratively, and users should return to this Action Item as needed to revise their plan.

## Discussion

The public-access D4DS Planner (d4dsplanner.com) is the first digital tool that supports transdisciplinary research teams in learning about and applying D4DS principles and the Fit to Context Framework in their work. Developed through a participatory design process, the two main components of the tool are the Education Hub, which contains resources to help users learn about D4DS, and the Action Planner, to support the application of D4DS principles and the conceptualization and design phase objectives of the F2C Framework.

The use of a participatory design approach engaged users in co-designing a D4DS Planner that incorporated users’ experiences and met researchers and practitioners’ needs and preferences.(23) Users indicated that the tool would be helpful in improving understanding of D4DS, enhancing co-design processes with partners, and ultimately would improve the effectiveness and sustainability of their products. Not surprisingly, features prioritized by the users were those that support learning about D4DS as this is a new and expanding area, including links to resources, and the process of engaging and collaborating with partners, including a roadmap tailored to a specified project to guide users in conducting D4DS. Overall, users liked the visual presentation of the tool and the actionable content that allows for developing a plan for dissemination and sustainability. The relative complexity in navigating the tool and having too many words in some sections were areas that users recommended modifying to enhance usability. While the participatory design approach was time intensive, the D4DS principles were new to users and time was necessary to solidify how our planner would be useful to end-users. Through this process, we learned that inclusion of users throughout the prototype build phase would have been beneficial, rather than waiting until the end to gain feedback on usability. This more iterative approach may have saved us time and financial resources by reducing the number of changes necessary after usability testing.

The D4DS Planner is designed for researchers to engage key partners throughout the process of planning a research initiative which is critical to ensure that products are designed to match contextual characteristics (i.e. priorities, needs, resources) of partners.(5, 6). Community engagement is critical to improving dissemination and sustainability by better aligning research activities with the priorities, needs, and assets of the intended users and local context.(5, 35) Several tools have been developed to guide conducting community-engaged research;(36) however, to our knowledge none have focused on engaging related to product development, dissemination, and sustainability.(37–40) Engaging the intended audience, particularly marginalized populations, in product design may promote health equity by developing interventions and products that meet the needs of historically marginalized populations and account for the their context. Intentionally selecting partners that do not create, reinforce, or maintain existing inequities is important if the process is intended to promote health equity. (41) When done well, co-creating ways of sharing and sustaining the intervention may increase reach, adoption, and sustained impact among diverse populations and marginalized groups. Our goal was to support teams in using the interactive features of the tool and D4DS methods with intention and equity in mind, with the goal of improving dissemination and sustainability outcomes and impact.

The D4DS Planner web tool has several potential uses for projects in different phases from conceptualization to sustainability. This tool supports users and their collaborators to deepen their understanding of the issue and work together as a team to identify and refine the solution (i.e., product) that effectively addresses a health problem that is important to the intended audience. Additionally, this tool can serve a crucial role in formulating comprehensive dissemination and sustainability plans. Early and ongoing planning for dissemination and sustainability enables the consideration of the intended audience and capacity and resources of the implementers and their contexts to enhance translation and utilization of research products in practice.(6) Further, the Action Plan generated from the D4DS tool can support the development of grant proposals and grant dissemination plans.

There are several limitations to this work. The target users for the D4DS Planner are research teams conducting clinical and public health-related research including researchers, practitioners, and community-based partners. However, we did not include community-based partners in our design sessions or usability testing. Subsequent usability testing will elicit perspectives from community-based partners to refine and tailor the tool to further support engagement with community partners. We also did not test the tool with collaborative teams (i.e., a researcher and community partner reviewing the tool together) which limited our ability to assess the tool’s features and functions that were designed to facilitate collaboration and engagement.

Usability testing was conducted on key features of the tool, but users did not comprehensively test the entirety of the tool to develop a D4DS action plan. Additionally, while our cues to equity were developed within the participatory design process, the ability of teams to easily use the cues in their research projects, whether cues have the intended impact on how the D4DS process is conducted, and the impact on health equity outcomes has not been tested. Future work will focus on usability testing among research teams, including community partners, and examine whether the tool and cues to equity were used as intended, as well as the impacts of using this process. The tool is limited to the English language, which will limit the use of the tool globally. Future iterations of the tool will consider building in a translation feature to broaden access to diverse populations.

Participatory design is an iterative and ongoing process.(23) The D4DS Planner is in its early iteration, and we are committed to refining the tool based on feedback from users representing diverse backgrounds, including researchers in non-academic and low resource settings and community-based partners. These varied perspectives are crucial, aligning with the tool’s primary goal of engaging a broad spectrum of partners in dissemination and sustainability planning to enhance product-context fit and ultimately promote health equity. To evaluate the tool’s impact, we will assess its reach and engagement using google analytics (e.g., number of users who access the tool, duration of use, completion of tasks) and its effectiveness and context-dependent implementation through user case examples. We will also design studies to test whether facilitation strategies are needed to support use of the tool; with a particular focus on understanding whether research teams without a D&I scientist are able to effectively use and complete the tool without facilitation.

## Conclusions

Dissemination and sustainment are critical to maximize the impact of research on health and well-being. The D4DS Planner can help research teams apply the Fit to Context Framework for dissemination and sustainability, facilitate partner engagement in research initiatives, and enhance the overall impact of their work. To our knowledge this is the first tool of its kind that facilitates engaging partners in product development, dissemination, and sustainability.

## Figures and Tables

**Table 1:** Overview of Design Process and Results

Exercise	Description	Overall Design Impact
**Design Session 1**		
1.1	how the tool could benefit and empower users	Goal of tool: Increase the transparency of D4DS and accessibility to multiple audiences, and foster collaboration. Primary users of the tool: researchers (with various level of D&I knowledge/background) 35 potential features
1.2	who might benefit from using this tool	
1.3	persona creation and value proposition generation exercise	
1.4	Free listing possible features	
1.5	Prioritizing features	
**Design Session 2**		
2.1	Kano Methodology^2^	13 features selected Key content areas: dissemination; education/literature; grant language; partner engagement; sustainability planning; methods
2.2	free list any other feature ideas and identify their favorite feature from the Kano survey	
2.3	grouped features into content areas for learning	
2.4	prioritized the most important content areas	
**Design Sessions 3–5**		
	iteratively envisioning and creating wireframes of the features that were prioritized	Wireframes for 1) account creation, 2) a questionnaire that would allow users to set-up a project and guide them on how best to use the tool, 3) a roadmap or visual process that walks the users through the D4DS process, 4) and the landing page.

1 Persona creation is defining different user types that might use our tool and how they may interact and benefit from the tool.

2 Kano Methodology is a survey used to prioritize potential features. (28, 29) The model classifies features as Must-have (i.e., I expect it and would be dissatisfied without it), One-dimensional (i.e., I expect it), Attractive (i.e., I like it), and Indifferent (i.e., I’m neutral).

**Table 2:** Kano Survey Results

Classification	Feature/Functionality
**Must-have** A user expects this feature to be implemented and they would be dissatisfied if the feature was not available	prominent links to other resources
**Attractive** A user may or not expect this feature but it would make them satisfied if it is implemented	**a step-by-step guide to applying D4DS (aka roadmap)** **a questionnaire that guides users to the content** **template of language for funding proposals** **a template of how best to engage with partners** **a fillable form of a research plan** **methods at each stage of the research process** **an account to save a user’s work** **search for examples of grant section, case studies** **customizable figure of a common model/theory/framework** search methods within topics and each stage of the design process allows users to contribute content (e.g., case examples) testimonials about users’ experiences a blank roadmap that could be used during partner engagement a tab specifically for community partners provides images/icons for infographic development an interactive social network map of users of this tool
**Indifferent** A user has a neutral opinion about whether a feature is implemented	short video presentations showing engagement methods or examples **opportunities to provide feedback via a ‘contact us’ interfac**e **has a large list of dissemination and sustainability literature** **allow users to search a database for references/literature by topic** its twitter account so users can interact with developers of the web tool and with other users an embedded twitter account opportunities to provide feedback via a pop-up survey

* no features classified as one-dimensional

* bolded items indicates those that were ultimately included in the tool.

**Table 3:** Summary of think-aloud usability testing results

Prompt	Academics	Practitioners	Experts
What works well in the D4DS web tool?	Academics liked that the D4DS Planner was action oriented and flexible to meet their project needs. Academics felt supported in this process by key features in the tool (e.g., key definitions, guidance questionnaires).	Practitioners liked how clear it was to use the different components of the tool including the account creation/login, navigating the landing page and action planner, and project creation. The clarity was enhanced by the clear instructions on how to use the action planner and ease of finding information. Practitioners also liked how the web tool is visually appealing.	Users liked the visual aesthetic of the tool, felt it was organized in a logical manner, and believed there were good resources that supported the user in how to use the web tool.
What are the significant pain points in the D4DS web tool?	Locating features and clarity on where to go within the D4DS tool was challenging. Setting up the project required additional clarity and parsimony.	The three major pain points for practitioners were the delay in receiving the verification email, difficulty in answering some questions in the project set up (i.e. budget, main objectives) and that some parts of the tool were wordy (i.e. guidance).	The layout of the pages can be challenging because the user does not know to scroll down. There is tension between layperson language and jargon. There is too much language in general throughout the web tool, although users asked for directions to guide their interactions.
What changes do users want to see in the D4DS web tool?	More guidance, tutorials, and examples to support users with get started and how to use the tool were requested. The use of simple language and project set-up requirements were a priority.	More information about setting up an account and project is needed. Reducing the amount of wording in the guidance section of the tool could enhance the readability.	Many of the changes have to do with the content or the jargon of the field. This presents a challenge. Because it is a specialized field and the point of the tool is to teach the language of the field, in addition to planning for projects.
General usability comments	The D4DS Planner pushes teams to think critically about aspects that are not commonly considered from the early stages of a project (dissemination and sustainability).	The tool seems more tailored to academics than practitioners with regards to its perceived use and functionality (e.g. account set up). Practitioners tend to be working with already designed products to implement and sustain, however the tool seems to provide support more towards designing a new product. The tool could also skip the email verification process like other web tools.	Users seemed to engage with the tool easily and noted that the web tool was visually appealing and professional looking.

**Table 4.** Perceived usefulness and appropriateness of the D4DS Planner

Question^1^	Mean(SD) (n=7)
The information the tool provides is useful.	4.3 (0.8)
The tool seems possible to use in my work.	4.4 (0.8)
The tool would help my work be more impactful.	4.1 (0.7)
The tool would help my work to reach a more diverse group of people.	4.0 (0.8)
The tool would make the information I want easier to access.	3.7 (1.1)
Overall score	4.1 (1.1)

1 Questions were asked on a 5-point Likert scale (strongly disagree to strongly agree)

## Data Availability

The datasets used and/or analyzed during the current study are available from the corresponding author on reasonable request.

## Abbreviations

AL: Allison LHotta PhD, OTR, OTR/L
BM: Brad Morse, PhD
D&I: Dissemination and Implementation
D4DS: Designing for Dissemination and Sustainability
DE: Design Exercises
F2C: Fit to Context Framework
MMK: Maura M. Kepper, PhD
SUS: System Usability Scale
TS: Thembekile Shato, PhD, MPH
UCD: User Centered Design
